# Fetal Death Due to an Unusual Coexistence of Two Umbilical Cord Anomalies: Analysis in a Forensic Perspective

**DOI:** 10.3390/diagnostics15111423

**Published:** 2025-06-03

**Authors:** Alice Ferretti, Maria Paola Bonasoni, Benedetta Petrachi, Giuseppina Comitini, Immacolata Blasi, Arianna Giorgetti, Paolo Fais, Susi Pelotti, Lorenzo Aguzzoli

**Affiliations:** 1 Unit of Obstetrics and Gynecologic Oncology, Azienda USL-IRCCS di Reggio Emilia, 42122 Reggio Emilia, Italy; alice.ferretti@ausl.re.it (A.F.); benedetta.petrachi@ausl.re.it (B.P.); giuseppina.comitini@ausl.re.it (G.C.); immacolata.blasi@ausl.re.it (I.B.); lorenzo.aguzzoli@ausl.re.it (L.A.); 2 Pathology Unit, Azienda USL-IRCCS di Reggio Emilia, 42122 Reggio Emilia, Italy; 3 Department of Medical and Surgical Sciences, Unit of Legal Medicine, University of Bologna, 40126 Bologna, Italy; arianna.giorgetti@unibo.it (A.G.); paolo.fais@unibo.it (P.F.); susi.pelotti@unibo.it (S.P.)

**Keywords:** umbilical cord true tight knot, furcate umbilical cord insertion, long umbilical cord, myonecrosis, stillbirth, post-mortem interval, intrauterine retention

## Abstract

**Background and Clinical Significance:** In stillbirth, bereaved parents must be promptly taken in by healthcare staff, and their requests to understand what happened must be fully explained and discussed. Empathic and open communication with the parents is fundamental in avoiding time-consuming lawsuits for investigating medical liability. Herein, we describe a case of stillbirth in which many elements exemplify potential concerns, either from a parental or judicial context. All these hypothetical questions will be addressed and discussed. **Case presentation:** A female stillbirth was vaginally delivered at 41 weeks of gestation after induction of labor. The baby was normally grown for gestational age, and the umbilical cord examination disclosed a length of 90 cm (75 cm attached to the placenta and 15 cm to the fetus), two tight knots, and a furcate insertion into the chorionic plate. Histologically, non-occlusive luminal thrombosis was found in the umbilical vein, extended to the cord insertion, and was in a few chorionic vessels. The knots showed myonecrosis in the vascular wall, widespread in the first one and more focal in the second, indicating prolonged contraction. **Conclusions:** The case we described, though fully diagnostically explained, raised many hypothetical questions that might have been brought up either in a judicial context or during communication with the parents of the autopsy results. Frequent questions may include what the cause of death was, when the baby died, if the death might have been avoidable, and, in the latter, potential medical liability.

## 1. Introduction

Umbilical cord true knots (UCTKs) have been reported with a low incidence among deliveries, ranging from 0.3 to 2% [1]. UCTKs usually take place between the 9th and the 12th week of gestation, when there is an abundance of amniotic fluid and the fetus is free of movement in the uterus in a relatively wide space. The development of a UCTK relies on the cord length, fetal size, and activities, which allow loop formation, cord twisting, and then knotting [2].

A known risk factor is a long umbilical cord, more than 60 cm, which is considered the normal length at term. Smoking in pregnancy, especially in the first months, favors the cord growing and lengthening. Other contributing factors include polyhydramnios and a small-for-gestational-age fetus [2]. Maternal conditions such as gestational diabetes, pre-eclampsia, previous miscarriages, and obesity may be associated with an increased risk of UCTK. The latter is more frequently found in male fetuses, which seem to be more generally active [3]. Fetal movements, in utero or during labor, may determine knot tightening with blood flow impairment. Fetal distress, a low Apgar score at birth, cerebral palsy, and stillbirth are different outcomes according to the severity of cord torsion and tightness [2,4,5]. UCTKs are usually with a single loop, but they can be double or with more complex morphology, especially in twin pregnancies [6,7]. Two knots in the same cord have been scarcely reported, but in all cases, perinatal complications were almost absent [8,9,10,11,12,13].

Herein, we describe a case of a female stillbirth at 41 weeks of gestational age in which two UCTKs coexisted in a 90 cm cord with furcate insertion into the chorionic plate. The autopsy and placental findings, cause of death, and estimation of the time of fetal death, with an analysis of post-mortem interval (PMI), are presented. These findings are then reviewed from a forensic perspective while also considering potential medical liability.

## 2. Case Presentation

### 2.1. Clinical History

The mother was a 31-year-old gravida 1, para 0, with no relevant past medical history. Her pregnancy was unremarkable; she never smoked and regularly attended all the scans planned. At 41 weeks of gestational age, during a scheduled ultrasound (US), planned as the standard of care for the third trimester, intrauterine fetal death (IUFD) was diagnosed. The US was performed at 15:00, and the mother reported reduced/absent fetal movements since the early afternoon of the day before (about 24 h before the detection of IUFD). The mother was admitted for induction of labor, and a macerated female baby was vaginally delivered after two days at 23:08 (56 h from the US and 80 h after the reported reduced/absent fetal movements).

### 2.2. Fetal Post-Mortem Examination

**Figure 1:**
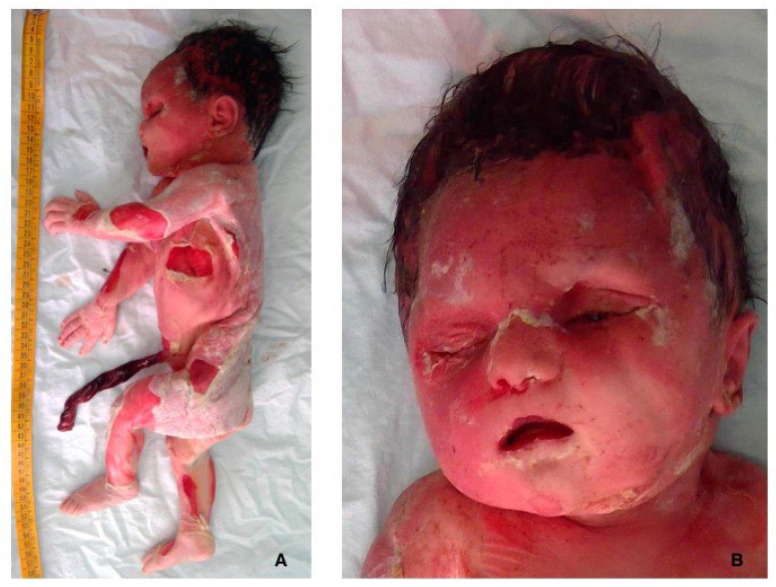
Fetal grade III maceration: at gross examination, the baby showed diffuse skin slippage (>5% of the body surface) and reddish cutaneous discoloration (**A**); and initial skull bone overlapping and sunken eyes were also observed (**B**).

The autopsy was performed two days after the delivery at 10 a.m. (about 91 h from the claimed reduced/absent fetal movements). The post-mortem external examination showed diffuse skin slippage (>5% of the body surface), reddish cutaneous discoloration, joint laxity, initial skull bone overlapping, and sunken eyes (Figure 1).

No dysmorphic features were observed. The baby weighed 3026 g and measured 49 cm in crown–heel length. The other measurements, compatible with 41 weeks of gestation, were as follows: crown–rump length 35 cm; foot length 7.5 cm; and head, chest, and abdominal circumference 31 cm, 29 cm, and 25 cm, respectively [14]. The fetal umbilical cord measured 15 cm in length. The internal examination revealed moderate bilateral pleural and abdominal bloody effusions. All the organs were brownish but anatomically normal. The autopsy findings overall corresponded to a grade III maceration [15]. Histologically, there was a widespread loss of nuclear basophilia [16]. In particular, nuclear basophilia was lost in the heart, liver, and kidneys.

### 2.3. Placental Examination

**Figure 2:**
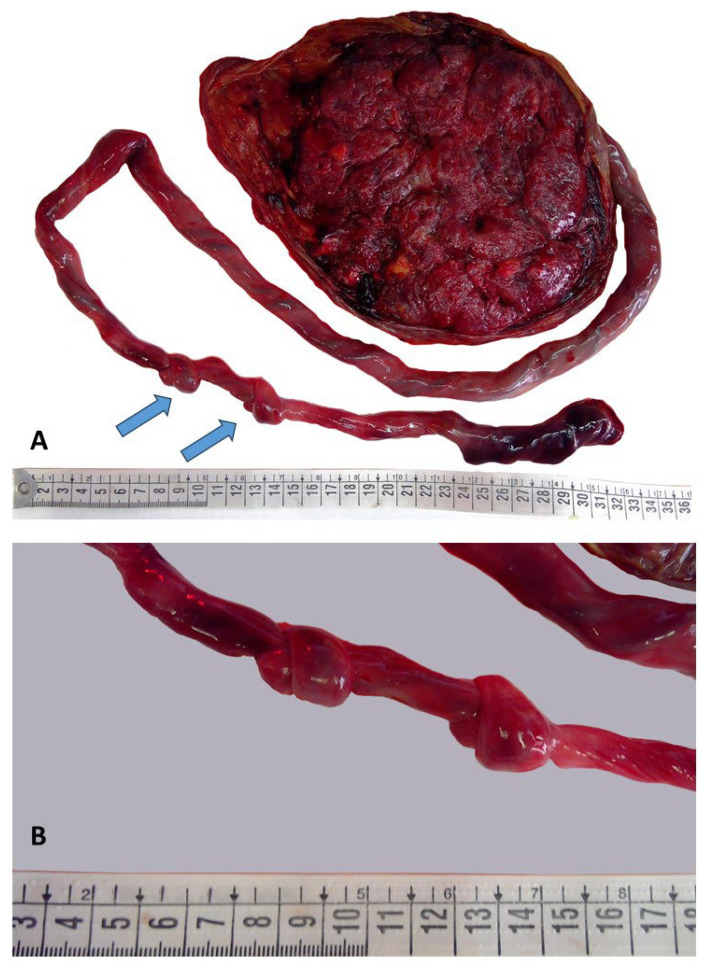
Two tight proper knots of the umbilical cord: the cord showed two knots, distanced 3 cm apart ((**A**), arrows). The first knot appeared more tightened ((**B**), knot on the left).

The placenta was received fresh and complete; it weighed 415 g (trimmed) and measured 16 × 16 × 2 cm. The three-vessel cord measured 90 cm in length with a coiling index of four coils/per 10 cm. Along the course of the cord, there were two tight knots, distanced 3 cm apart, located at 48 and 51 cm from the placental insertion, respectively (Figure 2).

**Figure 3:**
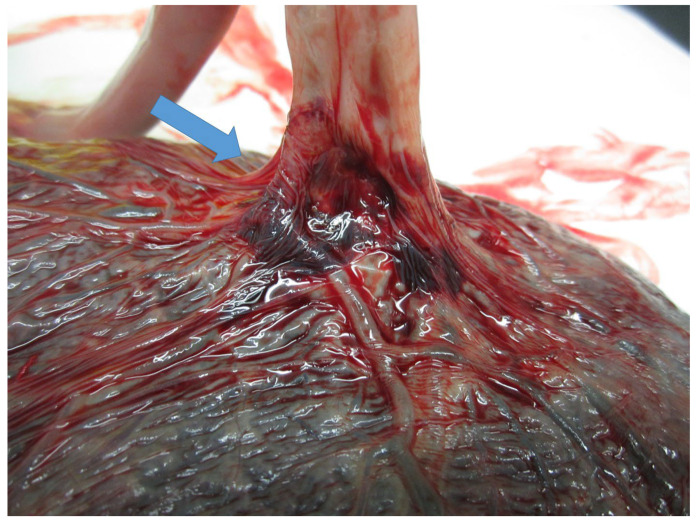
Umbilical cord furcate insertion: the umbilical vessels separated before reaching the placenta, losing the surrounding Wharton’s jelly (blue arrow).

The insertion into the chorionic plate was furcate and paramarginal (Figure 3).

**Figure 4:**
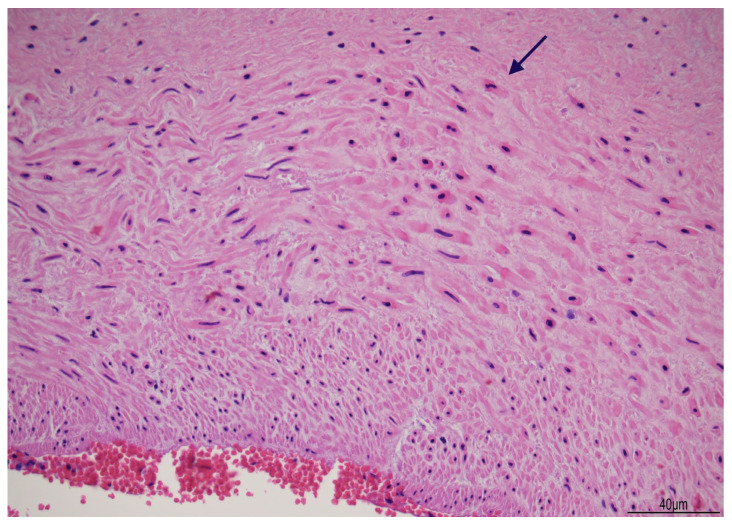
Vascular myonecrosis: within the knots, the wall of the umbilical cord vessels showed smooth muscle myonecrosis with eosinophilic cytoplasm and pyknotic nuclei (arrow) (Hematoxylin and Eosin, 20HPF).

Histologically, the umbilical vein, close to the placenta, showed non-occlusive thrombosis within the lumen, extending to the cord insertion into the chorionic plate. A few chorionic vessels also presented non-occlusive luminal thrombosis. In the first knot, the vascular walls displayed diffuse myonecrosis (Figure 4), but in the second knot, this damage was more focal.

**Figure 5:**
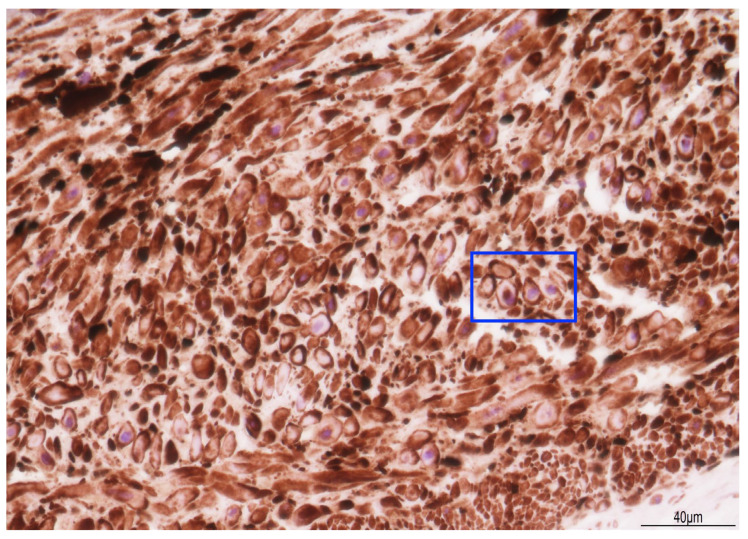
Immunohistochemistry for caldesmon: in the knots, cytoplasm staining was lost in most of the smooth muscle cells (rectangle, particular) (40HPF).

Immunohistochemistry for caldesmon exhibited a loss of staining in the cytoplasm (Figure 5).

The chorionic plate also showed focal subchorionitis with occasional granulocytes (maternal inflammatory response stage 0/4, grade 1/2) [17].

**Figure 6:**
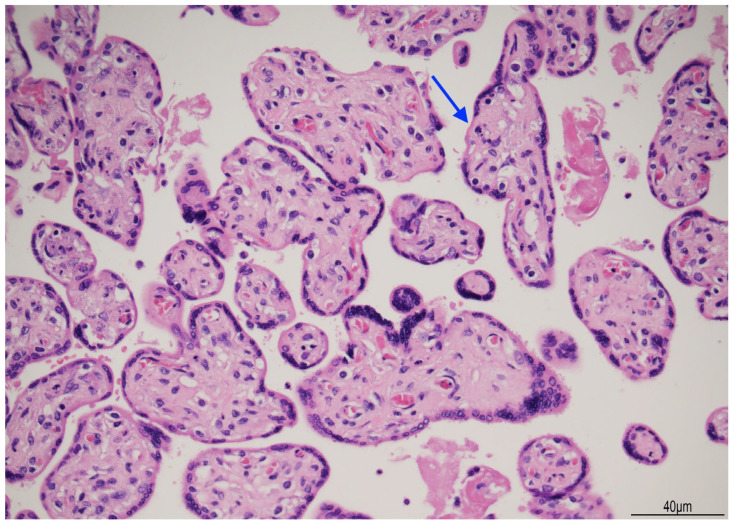
Placental parenchyma: most of the villi were dysmature with scarce vasculo-syncytial membranes. Karyorrhexis was also present (arrow) (Hematoxylin and Eosin, 20HPF).

In the placental parenchyma, villi were dysmature with focal karyorrhexis (Figure 6).

## 3. Discussion

The case of stillbirth we described, although unusual, is exemplary of many potential questions that might have arisen either from the parents, during the explanation of the autopsy results, or in a judicial context, for potential medical liability. Therefore, all the hypothetical questions will be addressed in a step-by-step approach.

### 3.1. The Cause of Fetal Death

First, regarding the cause of death, IUFD had been the consequence of the unfortunate combination of cord anomalies: a long cord (90 cm: 15 cm, connected to the fetus, and 75 cm, attached to the placenta), two tight UCTKs, and the furcate insertion. To the best of our knowledge, this coexistence has never been reported.

The umbilical cord connects the fetus to the placenta, carrying oxygen and nutritional elements and removing waste products. It is composed of two arteries and a vein surrounded by Wharton’s jelly. Anatomical abnormalities of the cord may compromise the blood flow, leading to fetal morbidity and mortality [2].

A long cord is a recognized risk factor for knot formation. The cord grows along with the fetus and the placenta, and its length is proportional to the gestational age. At term, the normal length is around 60 cm [2]. UCTK development occurs in the first trimester when the fetus can freely move in a wide intrauterine space. A long cord may form a loop through which the fetus can turn and flip; however, the likelihood of forming is low. However, the likelihood of forming two or more knots is extremely low [18].

US detection of a UCTK is possible through 2DUS in which the “hanging noose” sign can be recognized as a transverse section of the cord enfolded by a loop of the cord itself. An UCTK detection rate in prenatal 2DUS is low, but it can be increased with a 3DUS and 4DUS. A color Doppler US can be added to identify knot tightening. However, false-negative scans can be due to fetal position that can hide the knot or maternal habitus. False-positive scans can also be attributed to a twisted or conglomerated course of the cord [19]. Although a prenatal diagnosis of UCTKs is possible, their management has not been focused on by the International Society of Ultrasound in Obstetrics and Gynecology (ISUOG) and other groups [20]. In a prenatal setting and routine US screening, there is no indication in the study of the cord and knots [20]. Although some studies recommend a close follow-up during pregnancy and even a planned birth at 37 weeks of gestation [19], others are more conservative, avoiding excessive interventions and limiting management to a “wait and see” assessment up to the labor [3].

In a recent review and meta-analysis by Hayes et al. [4], the risk of stillbirth in the presence of a UCTK was highlighted by an odds ratio (OR) of 3.96 (95% confidence interval) based on data from seven studies comprising 930,314 births. This finding strengthened the association between UCTKs and fetal demise. However, the overall incidence of stillbirth was relatively low compared to the total study population, including live births with an UCTK, as shown in Table 1.

As already mentioned, a long cord favors knot formation. In our case, the cord measured 90 cm in total length, and there were two tight knots. Both knots displayed myonecrosis in the vascular wall, though in one, the damage was more extensive.

Myonecrosis has been described in vascular prolonged contraction, in which the depletion of metabolic resources leads to the mechanical destruction of the myofilament apparatus [26]. In our case, caldesmon immunohistochemistry revealed a loss of cytoplasmic staining, likely indicating a disruption in the actin–myosin complex. Caldesmon is a protein activated by the cytoplasmic calcium concentration: at low levels, it keeps the muscle relaxed, and at higher levels, it determines muscle contraction [27]. In the two knots, the prolonged vascular traction and the local attempt to loosen them had probably determined the cellular damage. The two knots were tight, and blood flow impairment was confirmed by endoluminal thrombosis in the umbilical vein course close to the placenta, in the chorionic cord insertion, and in some chorionic vessels.

In our case, UCTKs were not identified prenatally, despite the planned maternal scans, but the cord was not specifically assessed as not included in the current practice [20]. In addition to UCTKs, in the case we presented, the cord had a furcate insertion into the chorionic plate. A furcate cord is the branching of the umbilical vessels before insertion into the placenta, leaving them devoid of Wharton’s jelly. This condition favors adverse effects, such as vascular compression, rupture, and thrombosis, increasing the risk of IUFD. The incidence of furcate insertion has been estimated as 0.1% of all deliveries, but the true incidence, despite US-feasible prenatal detection, is likely understated [28,29].

Although unusual, this case of stillbirth was fully explained from a pathological and diagnostic point of view. The parents were received, the cause of death was carefully described, and the element of “unpredictability” was pointed out. It was also clarified that umbilical cord knots are not easy to detect during an US, and the current clinical practice does not recommend specific cord assessment [20]. The parents were satisfied with the explanation and no further requests were pursued, accepting the fact that UCTKs, even detected during an US, can undergo sudden, inevitable tightening. Especially the latter element soothed their questions as, “should we have noticed anything to avoid it and hurry to the hospital?”. Effective communication from the healthcare staff to the grieving families can be paramount in avoiding unnecessary lawsuits, mainly undertaken in order to better understand what caused IUFD. Although emotionally difficult, adverse outcomes must be straightforwardly discussed with the families, and their concerns openly addressed by health professionals [30,31]. On the other hand, if the parents had claimed compensation or medical liability evaluation, it would have been demonstrated that the current clinical practice was respected, as the mother accurately attended the US plan, and, currently, there is no specific requirement for cord assessment [20]. Moreover, knot tightening was sudden because the fetus was not growth-restricted and the cord blood flow was not compromised during pregnancy. Therefore, the adverse event was “unpredictable” and “unavoidable”.

### 3.2. The Time of Fetal Death

The second difficulty to solve is to identify when the baby died. Unless the fetus was instrumentally monitored, and the time of death (TOD) is therefore more precise, it is challenging to ascertain with accuracy the exact moment of demise. According to the current literature, the only methods available are those proposed by Genest et al. [16,32,33], in which the extent of external maceration, histological loss of nuclear basophilia, and placental examination may help to outline the period of intrauterine retention and the interval between death to delivery. In stillbirth, identifying the TOD, or at least circumscribing it as much as possible, is crucial in case of alleged medical malpractice, as the time of fetal demise must be accordingly evaluated with healthcare assistance. If the fetal death occurred before or after hospital admission, the whole process of allegations may significantly change [34]. In our specific case, the mother underwent a routine ultrasound (US) in which IUFD was detected. The examination was at 15:00, and reduced/absent fetal movements were reported at least 24 h before. Labor was then induced, and a macerated female fetus was spontaneously delivered after 2 days at 23:08, counting 56 h since the US and about 80 h since the reported reduced/absent fetal movements. The autopsy was carried out at 10:00, almost 2 days after the delivery (about 91 h since the claimed absent/reduced fetal movements). The post-mortem external examination showed diffuse skin slippage (>5% of the body surface), reddish cutaneous discoloration, joint laxity, initial skull bone overlapping, and sunken eyes. According to Genest et al. [33], the extent of maceration would correspond to a death-to-delivery interval of ≥ 18 h. Histologically, loss of nuclear basophilia was thoroughly detected, especially in all cells of the liver and the whole myocardium. These findings would extend the death-to-delivery interval between 48 h (heart) and up to 96 h (liver) [16]. Considering the estimated death-to-delivery interval, which was 80 h, the histological finding of a diffuse loss of nuclear basophilia in the liver would fit within the potential interval of a minimum of 80 h up to 96 h. Although the fetus was correctly kept refrigerated, putrefaction was slowed down but not completely stopped [34]. Therefore, the delay of almost 2 days in post-mortem examination and organ sampling might have further contributed to the autolytic changes such as skin epidermolysis and a widespread loss of nuclear basophilia.

In fact, the studies by Genest et al. [16,32,33]—the only currently available ones—have the limitation that they do not take into account the delivery-to-autopsy interval, rather only stating that the fetus was kept refrigerated at 5 °C [34].

### 3.3. The Type of Death

The third and last question is the type of death—if it was acute or more subacute/chronic. First of all, it must be considered that the fetus was retained in utero for at least 56 h, up to 80 h, adding the time of the maternal reporting of ceased fetal movements. Therefore, the effects of intrauterine maceration and the delayed autopsy interval must be taken into account. However, at post-mortem, despite maceration, the fetus did not show features of acute asphyxia, such as residual cutaneous meconium staining, pleural/pericardial petechiae, or endoalveolar keratinic squames and/or meconium indicative of gasping [35]. In this specific case, it was more likely that knot tightening had progressively started, and vascular thrombosis might have had the time to form, determining a more prolonged agonic interval and inducing a subacute/chronic type of death.

### 3.4. Minimally Invasive Tissue Sampling in a Forensic Context

In our specific case, we performed a complete and thorough diagnostic autopsy, including fetal external and internal gross examinations, histological sampling, and placental analysis. The results were, overall, exhaustive and easily applicable both for diagnostic and/or forensic purposes. Especially during the COVID-19 pandemic, limited autopsies with minimally invasive tissue sampling (MITS) came up as an alternative diagnostic tool in order to limit the least possible professional exposure to a hazardous disease [36,37]. However, in a forensic context, MITS is scarcely applicable, as a forensic autopsy must provide as much data as possible. A thorough forensic autopsy must contribute to explaining the following inquiries: identifying the cause and manner of death and the time of death (post-mortem interval) [38]. Moreover, MITS can reduce the amount of tissue sampling necessary for histological and/or toxicological purposes, significantly diminishing the amount of data valuable in a court of law. Unless there are specific conditions that impose MITS, for example, for staff personal protection, this practice should be avoided in a judiciary context.

## 4. Conclusions

The case of the stillbirth we described has offered many exemplary doubts, and the application of the medico-legal approach can be used in many other similar cases if parents’ doubts or judicial concerns develop.

In stillbirth, if requests for compensation arise, an estimation of the time of death is of paramount importance. Circumstantial data, such as the date and time of reduced or absent fetal movements, as monitored or reported by the mother; the date and time of hospital admission; and subsequent delivery must be fully registered and then easily accessible and retrievable. The date and time of the autopsy must also be recorded. Every time interval must be fully reconstructed in order to evaluate correct healthcare assistance and potential medical liability.

## Figures and Tables

**Table 1:** Cases of stillbirth with UCTK according to literature.

Reference	Stillbirth Cases/Total Number of Cases with UCTK	Percentage %	Incidence
Blickstein et al. [21]	2/54	3.7	0.037
Airas et al. [22]	4/288	1.4	0.014
Tantbirojn et al. [23]	2/33	6.06	0.06
Raisanen et al. [24]	3/339	0.88	0.009
Linde et al. [1]	At term: 83/1216	6.82	0.068
Linde et al. [1]	Pre-term: 58/2046	2.83	0.028
Gaikwad et al. [25]	2/3 case series	66.66	0.666

## Data Availability

The data presented in this study are available on request from the corresponding author.
